# Primary Leiomyosarcoma of the Male Breast: A Case Report

**DOI:** 10.1155/2010/534102

**Published:** 2010-10-27

**Authors:** Yazan Masannat, Haytham Sumrien, Yousef Sharaiha

**Affiliations:** ^1^Princess Royal University Hospital, Farnborough Common, Orpington, London, BR6 8ND, UK; ^2^Prince Philip Hospital, Llanelli, Wales SA14 8QF, UK

## Abstract

Primary leiomyosarcoma of the breast is a rare tumour with only around thirty cases reported in the literature. Most of the cases reported are in females, while only a few are reported in males. We present a case of primary breast leiomyosarcoma in a 59-year-old man that presented with a subareolar lump which felt to be benign clinically and radiologically but proven to be a leiomyosarcoma on excision.

## 1. Introduction

**Figure 1:**
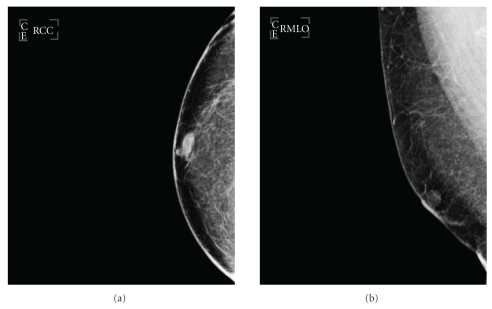
Mammography: Right breast CC & MLO Views: Isodense, well-defined lesion within the retroareolar area 18 × 13 mm in diameter.

A 59-year-old male patient was referred to the breast clinic with a breast lump underneath the right nipple. The lump was painless with no other associated complaints. On clinical assessment it was a firm, discrete benign feeling lesion, about 15 mm in diameter. There were no palpable axillary lymph nodes. On radiological assessment, both Ultrasound scan and Mammography showed a benign looking mass just lateral to the right nipple, with discrete well-defined edges (Figure 1). This measured 18 mm × 13 mm on ultrasound scan.

**Figure 2:**
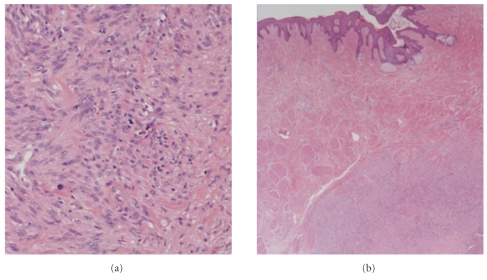
Histopathology section showing a spindle cell tumour with pleomorphism and mitoses. The figure on the right-hand side demonstrates also tumour cells with mitoses and paranuclear vacuolation.

The patient refused to have a core biopsy, therefore, an excisional biopsy was done under local anaesthesia. Histopathology showed a 20 mm spindle cell tumour with pleomorphism and mitoses (Figure 2). On Immunohistochemistry, the lesion was positive for Desmin and Smooth Muscle Actin (SMA) while it was negative for S100. All these were in keeping with primary leiomyosarcoma. The nearest margin was the superficial which was 4 mm from the tumour. Staging investigations were normal. Simple mastectomy without axillary dissection was performed and a residual disease was confirmed. The cavity wall confirmed the presence of a further 5 mm of similar tumour. A multidisciplinary team decision postoperatively was to keep the patient under regular followup with no need of any further adjuvant therapy. The patient has been followed up for 26 months without evidence of recurrence.

## 2. Discussion

Sarcomas comprise less than 1% of all primary breast neoplasms and only a minority of these are leiomyosarcomas [1]. Sarcomas in the breast have been described since the early 20th century but most probably the first confirmed case of leiomyosarcoma on IHC was reported in the late 1960s [2]. Though it was described as fibrosarcoma in the report, Munitiz et al. commented in 2004 that the pathological features on optical and electronic microscopy were in keeping with Leiomyosarcoma. It has been reported that only 25 cases in the literature are confirmed primary leiomyosarcomas of the breast proven on IHC or electron microscopy [3]. Confirming the diagnosis on either IHC or electron microscopy is important as it is sometimes difficult to differentiate these pathologically from poorly differentiated sarcomatoid carcinomas [1].

Diagnosis can be challenging because of the nonspecific clinical and radiological findings. Clinically they present with a progressively enlarging tumour. The presence of enlarged axillary nodes is rare making axillary node staging unnecessary [1, 4]. Radiological findings are also non specific. While some have reported a welldefined lesion that can be mistaken for benign pathology as in our case [1], others have reported malignant looking lesions [5]. Fine needle aspiration cytology can be challenging as these tumours can be mistaken for poorly differentiated carcinoma, metaplastic carcinoma, or other sarcomas such as malignant phylloides [3, 6]. On histopathological assessment, Immunohistochemical staining is essential as adjuncts to differentiate leiomyosarcomas from other tumours and soft tissue sarcomas. These tumours are usually positive for desmin, smooth muscle actin, muscle specific actin and negative for S100 (neural tumour marker), cytokeratins, and epithelial markers [1, 3, 7, 8].

The cornerstone of treatment is surgical excision. Most authors advice either radical or simple mastectomy arguing that wide excision is associated with higher rate of recurrence [1, 2, 7, 9]. Conservative surgery with wide local excision and clear margins has been described [3, 10, 11]. Though axillary node dissection is not necessary, it has been performed in many of the reported cases because of uncertain preoperative diagnosis or if there were clinically palpable nodes [5, 7, 10, 12]. Long-term followup is important as some of these tumours have been reported to recur more than 10 years posttreatment [13]. 

In summary, this tumour is a rare tumour that can be a diagnostic challenge to clinicians as it has no specific clinical or radiological features. This emphasizes the importance of triple assessment in all breast lumps, even if clinically and radiologically they are benign. The mainstay of treatment is surgical excision with clear margins and longterm followup is essential.
